# Multiple Infections with *Cardinium* and Two Strains of *Wolbachia* in The Spider Mite *Tetranychus phaselus* Ehara: Revealing New Forces Driving the Spread of *Wolbachia*

**DOI:** 10.1371/journal.pone.0054964

**Published:** 2013-01-23

**Authors:** Dong-Xiao Zhao, Da-Song Chen, Cheng Ge, Tetsuo Gotoh, Xiao-Yue Hong

**Affiliations:** 1 Department of Entomology, Nanjing Agricultural University, Nanjing, Jiangsu, People’s Republic of China; 2 Laboratory of Applied Entomology and Zoology, Faculty of Agriculture, Ibaraki University, Ami, Ibaraki, Japan; Wageningen University, The Netherlands

## Abstract

Cytoplasmic incompatibility (CI) has been proposed as a major mechanism by which certain strains of *Wolbachia* to invade and persist in host populations. However, mechanisms that underlie the invasion and persistence of non-CI strains are less well understood. Here, we established a spider mite *Tetranychus phaselus* population multiply infected by *Cardinium* as well as two distinct lineages of *Wolbachia*, designated *w*Con and *w*Ori, to study the forces driving the spread of the non-CI strain of *Wolbachia w*Ori. Interestingly, we found that *w*Ori provided a longevity advantage to its female hosts under ideal conditions, making *w*Ori stay longer in this population, and then being transmitted to more offspring. Furthermore, the lifespan of uninfected females was reduced when mated with multiple-infected males. As a result, the uninfected population is attenuated by the multiple-infected males. Thus, we infer that the host age effects of multiple infection may represent sufficient forces driving the spread of *w*Ori through the host population.

## Introduction

*Wolbachia* are obligate intracellular rickettsia-like bacteria that occur in numerous invertebrates. Infection with *Wolbachia* is found frequently in insects [1], mites [2], [3], spiders [4], [5], crustaceans [6], and nematodes [7]. Another maternally inherited symbiont *Cardinium* from the *Bacteroidetes* group has been found in four insect orders and approximately 6–7% of arthropods species that have been examined [8], [9]. These intracellular bacteria have recently received much attention for their various intriguing reproductive abnormalities that account for their success, including parthenogenesis, feminization, male-killing, and cytoplasmic incompatibility (CI). CI is the developmental arrest of insect embryos that result when females are mated with males that have a different infection status.

Several reports have noted that CI, the most common of the reproductive abnormalities, imparts the ability to *Wolbachia* to invade host populations [10], [11], [12]. This effect of CI has been quantified and modeled in several arthropod species including *Drosophila simulans* [11] and *Aedes albopictus* [13], [14]. Once enough individuals are infected, any disadvantages associated with infection are outweighed by the presence of CI-induced mortality. In other words, the more common the infection is, the more likely it is that uninfected females will encounter infected males and hence suffer reproductive losses. While CI has been proposed as a major mechanism allowing certain strains of *Wolbachia* to invade and persist in host populations, mechanisms that underlie the invasion and persistence of non-CI strains are less well understood. It is surprising, therefore, that spread mechanism of *Wolbachia* symbioses has not all been identified yet, especially in view of the fact that several *Wolbachia* strains have invaded natural populations even though they induce little to no CI [15]. Several reports show that the protection of their hosts from viruses and other pathogens has in recent years been revealed to be a major driving force in *Wolbachia* [16], [17]. It is hypothesized that these strains may provide an as yet undetermined fitness benefit to their hosts. To our knowledge, this hypothesis has not yet been tested.

The spider mite *Tetranychus phaselus* is a serious agricultural pest that damages many crops including cotton, bean, cucumber, eggplant, pepper, tomato, cucurbits, papaya, passion fruit. Infections of both *Wolbachia* and *Cardinium* in the same host are known in a number of species in the Acari and the Hymenoptera [8], [9], [18], [19], [20]. Moreover, a number of studies have reported that multiple infections of *Wolbachia* occurred commonly in several species, *Drosophila simulans* [21], [22], *Aedes albopictus* [23], *Nasonia vitripennis* [24], [25], *Callosobruchus chinensis* [26], [27], the *Drosophila* parasitoid *Leptopilina heterotoma* [28] and *Tetranychus cinnabarinus* (*T. urticae* red form) [29]. Recently, we found a multiple infection pattern in the spider mite *T. phaselus*. *Wolbachia*-superinfection (that is co-infection with two or more strains), and *Cardinium*-infection occur naturally in the same host. To our knowledge, this is the first case of multiple infection with *Cardinium* and two strains of *Wolbachia* in arthropods.

The aim of the present study was to discover the mechanism by which *w*Ori is spread in co-existence with *w*Con and *Cardinium* in *T. phaselus*. To this end, we established three lines of *T. phaselus*, an uninfected line (*Wolbachia*-free and *Cardinium*-free), a double-infected line (infected by *w*Con and *Cardinium*), and a multiple-infected line (infected by *w*Con, *w*Ori and *Cardinium*).We examined the levels of CI induced in *T. phaselus* by double-infection and multiple-infection, and compared the longevity, egg hatch, fecundity and bacteria densities in the three lines of *T. phaselus*. Our results reveal that *w*Ori has effects on host age that may act as new, non-CI forces for the spread of a *Wolbachia* strain.

## Materials and Methods

### Preparation of Spider Mite Lines

The spider mite *Tetranychus phaselus* was collected from soybean [*Glycine max* (L.) Merr.] leaves in Cixi, Zhejiang Province, east China in July 2009. Mites were reared on a leaf of the common bean (*Phaseolus vulgaris* L.) placed on a water-saturated sponge mat in Petri dishes (dia. 9) at 25±1°C, 60% r.h. and under L16-D8 conditions.

In order to cross infected and uninfected individuals, 100% double-infected, 100% multiple-infected and 100% uninfected lines were prepared for the population. One female from the teleiochrysalis stage was allowed to lay eggs without being crossed with males. The eggs were reared until adulthood (males). After the males reached sexual maturity, they were backcrossed with the mother. Then, the female adults were transferred to new leaf discs and were allowed to lay eggs for 3–5 days. A female was checked for *Wolbachia* and *Cardinium* infection status by PCR amplification. The eggs were separately reared on new leaf discs depending on the infection status of the mother. The above process was continued for three to four generations until a 100% infected population was obtained. The eggs of the uninfected mothers were reared to establish the uninfected line.

### DNA Extraction and Diagnostic PCR

In order to exclude the influence of different body sizes of female and male mites, DNA was extracted by homogenizing a single adult mite in a 15 µl (male) or 25 µl (female) mixture of STE buffer (100 mM NaCl, 10 mM Tris-HCl, 1 mM EDTA, pH 8.0) and proteinase K (10 mg/ml, 2 µl) in a 1.5 ml Eppendorf tube. The mixture was incubated at 37°C for 30 min and then 95°C for 5 min. The least bacteria DNA is lost with this method of DNA extraction.

To check for *Wolbachia* and *Cardinium* infection, all PCR reactions were run in 25 µl buffer using the TAKARA Taq kit (Takara Shuzo, Otsu, Japan): 16.3 µl H_2_O, 2.5 µl 10×buffer, 1.5 µl of 2.5 mM deoxyribonucleotide triphosephates (dNTPs), 1.5 µl of 25 mM MgCl_2_, 0.2 µl Taq polymerase (5 U/µl, Takara), 2 µl sample and 1 µl primers (10 mM each). The primers used for the detection of *Cardinium* were CLOf and CLOr [8], which amplified ca. 450 bp of 16S rDNA. Each PCR was run for one cycle at 94°C for 2 min, 35 cycles at 94°C for 30 s, 57°C for 30 s, 72°C for 30 s and a final extension of 5 min at 72°C. We investigated the *Wolbachia* infection status of 38 Chinese populations of agriculture mites, including *T. kanzawai*, *T. cinnabarinus*, *T. urticae*, *T. phaselus* and *Amphitetranychus viennensis*. The results suggest that all *Wolbachia* infected in Chinese populations of agricultural mites have been classified into subgroup Ori and Con belonging to supergroup B, according to the *wsp* gene sequences. In order to monitor the *Wolbachia* strains in those agriculture mites, we designed specific primers, *w*Con *–*F (5′-AAATGCAACAGGTAAAGAAAAGG-3′) and *w*Con*-*R (5′-CAAATCCTTTTTGATCTTTAACTGTA-3′), *w*Ori *–*F (5′-ACATATAAATCAGGTAAGGACAACA-3′) and *w*Ori *–*R (5′-CCAGATTTTTTATCACCAGTAACC-3′). Both of the *Wolbachia*-specific *wsp* primers were designed by Primer5 according to the *wsp* sequence of *w*Ori and *w*Con, which were deposited in GenBank (*w*Ori: AY585713;AY785372;AY785371; AY785376; AY585714; AY585712; AY785377; AY785374; AY585713; AY712955; AY712954; AY785375; AY785372; AY785371; AY785373; DQ016539; AB096218-AB096238; AF404766; AF217719; AJ437286; AJ437287; AJ437289; AJ437290; AF358417; AF348452; AF404765; AJ437288, *w*Con: DQ016535; DQ016536; DQ016534; AB096221-AB096223; AB096228; AJ437290; AF510085; AY596786), which amplified ca. 320 bp of wsp gene. Cycling conditions were 94°C for 2 min, followed by 35 cycles at 94°C for 30 s, 55°C for 30 s, 72°C for 30 s and a final extension of 5 min at 72°C.

For samples failing to amplify using *Wolbachia* and *Cardinium*-specific primers, primers par COI-forward and COI-reverse [30] were used to amplify mitochondria DNA as a positive control for template DNA quality. Approximately 7 µl of the PCR products were electrophoresed in a 1.0% agarose gel in TBE/EtBr for 40 min at 60 mA, and then photographed on a UV transilluminator. Amplified fragments were purified using a Gel Extraction Mini kit (Watson, Shanghai, China). Then, the distinct single-band amplicons were cloned into pGEM T-Easy Vector (Promega, Madison, WI, USA) and the positive clones were screened and sequenced directly. The sequence data helps us to confirm that the PCR positive individuals are infected with *w*Ori, *w*Con and *Cardinium*.

### Cross Experiments

To reveal how the different *Wolbachia* strains and *Cardinium* influenced CI modification and rescue, crossing experiments were performed (Table 1). Female teleiochrysalids, the last developmental stage before adult emergence, were placed with two males on the same leaf disk. We used 1-day-old virgin males produced as a cohort by groups of females isolated as teliochrysalids. This procedure was designed to avoid the potential decrease of the *Wolbachia* and *Cardinium* effects due to male ageing or repeated consecutive mating. Males were discarded 2 days after the females’ eclosion, and mated females were allowed to oviposit for 5 days. Eggs on leaf discs were checked daily to determine the hatchability, sex ratio (% daughters) and mortality of offspring. Fecundity was estimated as the total number of eggs laid in the first 5 days. Data were analyzed with one-way analysis of variance (ANOVA), and the means were compared using the Tukey-HSD test (SPSS 17.0). To normalize the data, log transformation was used for the number of eggs laid per female, and arcsine square root transformation was used for egg hatchability, sex ratio and mortality.

**Table 1:** Fecundity of females when they are mated with males on different infection statuses.

Cross F×M	N^a^	Number of eggs
U×U	17	24.0±1.2a
U×Iwc	25	19.0±0.6b
U×Iwwc	18	15.8±0.8c
F2, 57b		18.520***
Iwc×U	15	32.4±0.8a
Iwc×Iwc	13	20.1±0.8b
Iwc×Iwwc	20	22.5±1.1b
F2, 45b		42.926***
Iwwc×U	15	27.9±1.1a
Iwwc×Iwc	13	13.4±0.8c
Iwwc×Iwwc	15	23.6±0.8b
F2, 40b		62.226***

a Number of pairs tested.

b Means (±SE) differ significantly at *p*<0.05 (*), *p*<0.01 (**) and *p*<0.001 (***) (ANOVA); NS, not significant at the 5% level. Values in a column followed by different letters (a, b, or c) are significantly different at *p*<0.05 (Tukey HSD test).

### Bacteria Density Measurement

Bacteria densities were measured in the first 20 days of the life span of females and in the first 8 days of the life span of males (because of their shorter longevity and the higher escape frequency). Copy numbers of *w*Ori, *w*Con and *Cardinium* in individual mites were estimated by Quantitative PCR. Quantitative PCR was carried out with the ABI PRISM 7300 Sequence Detection System (Applied Biosystems). The 20 µl reaction mixture consisted of 10 ul 2×SYBRq Premix Ex Taq (Applied Biosystems), 0.4 µl 10 mM of each primer, 0.4 µl 50×ROX Reference Dye, 2 µl DNA template and 6.8 µl H_2_O in single wells of a 96-well plate (PE Applied Biosystems).

For the selective amplification of a small portion of the *Cardinium* 16S rDNA gene (133 bp) and *Wolbachia wsp* gene (*w*Ori, 137 bp and *w*Con, 112 bp), the following primers were designed and used: *w*OriQ-F (5′-GCA GCG TAT GTA AGC AAT CC-3′), *w*OriQ-R (5′-ATA ACG AGC ACC AGC ATA AAG-3′); *w*ConQ-F (5′-CTC GTT ACT TCG GTT CTT ATG GC-3′), *w*ConQ-R (5′-TTA AAC GCT ACT CCA GCT TCT GC-3′); cardQ-F (5′-CCT GGG CTA GAA TGT ATT TTG-3′), cardQ-R (5′-AAA GGG TTT CGC TCG TTA TAG-3′). PCR primers and probes were designed using Primer 5. The Q-PCR cycling conditions included 1 cycle (10 s 95°C) followed by 40 cycles (5 s 95°C, 31 s 60°C), and finally 1 cycle (15 s 95°C, 1 min 60°C, 15 s 95°C). Eight super-infected males of 1-, 4-, 8- and 12-day-old were collected. DNA of single mites was extracted using the above method. Three replicates were run and averaged for each DNA sample. Negative controls were included in all amplification reactions.

Standard curves were plotted using a 10-fold dilution series consisting of 10^−7^ to 10^−3^ dilutions of the DNA standards prepared from plasmid DNA. The quality and concentration of all purified standard DNA were measured by OD absorbance at 260 nm. The number of molecules in all samples was determined from the threshold cycles in the PCR based on the standard curve. Statistical analysis was performed using the Mann-Whitney *U*-test.

### Survival Assessment

Differences in host longevity were observed in comparisons of the different infection types. We measured age-specific survival of the females that are uninfected, single-infected and double-infected. For the infected females, we compared the longevity difference between single-infected and double-infected virgins. Meanwhile, the longevity of uninfected females was also measured. They are divided into four groups, according to their different mating patterns, including virgins as well as those mated with uninfected, single-infected and double-infected males. In order to test the longevity of virgins, 50 teleiochrysalids (the last developmental stage before eclosion) were placed on the different leaves according to the infection patterns. The new adults emerging within 24 hours were tested for longevity. For the longevity of mated females, every newly emerging adult was separately placed with two males with different infection statuses (uninfection, double-infection and multiple-infection) on the same leaf disk. The males were allowed to stay with females for 48 hours, which was designed to avoid the variance in mating times. Leaves were monitored every day, and dead females were removed and counted until all females had died. Survivor curves for individual hosts were compared using the Kaplan-Meier log-rank test (SPSS 17.0).

## Results

### PCR Detection of *w*Ori and *w*Con in Spider Mites

Thirteen out of forty mites in a rearing group were positive in PCR assay for *Wolbachia* infection, among which four were infected only by *w*Con and nine were superinfected by both *w*Ori and *w*Con. Moreover, we detected *Cardinium* 16S rDNA in all the*Wolbachia* positive mites. However, we did not discover any individuals singly infected with *Cardinium, w*Ori or *w*Con. As a result, we established 3 lines including a 100% uninfected line (U), a 100% double-infected line (infected with *w*Con and *Cardinium*, Iwc) and a 100% multiple-infected line (infected with *w*Ori, *w*Con and *Cardinium*, Iwwc).

### Effects of *w*Ori Effects on Host Reproduction

In order to clarify the effect of wOri on host reproduction, four crosses between Iwc and Iwwc were conducted.

**Figure 1:**
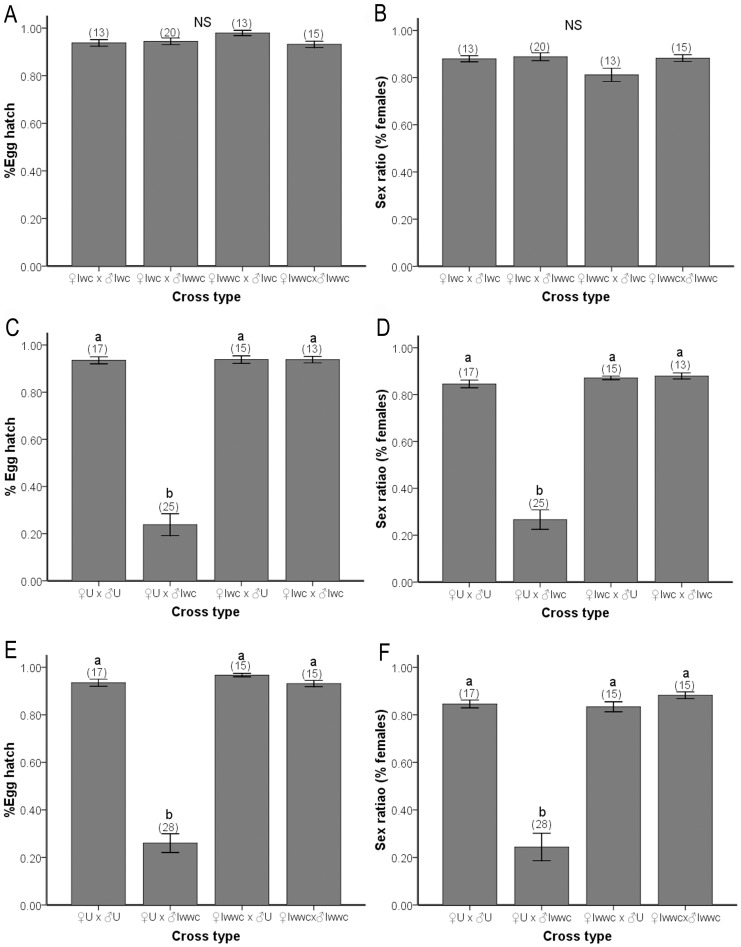
CI resulting from crosses of the uninfected, double-infected and multiple-infected strains. Results are mean percent embryo hatch ±SEM and sex ratio ±SEM. Number of replicates for each of the nine cross types are shown in parentheses. a and b represent statistic groups (Tukey-HSD test, p<0.05); NS, not significant at the 5% level.

The hatchability of eggs was compared among the four crosses in which the CI of *w*Ori was investigated (Fig.1. A, B). No significant differences were found between the predicted incompatible cross (Iwc×Iwwc) and the other compatible crosses for any of the investigated traits. We found that males extra infection with *w*Ori (Iwwc) were not able to induce CI with single-infected females (Iwc). Therefore, under this intricate infection status, *w*Ori existed as a non-CI strain in this spider mite population. Meanwhile, CI expression of double-infected (Iwc) and multiple-infected (Iwwc) males was also contrasted. There was no significant difference in hatchability and sex ratio between the two predicted CI crosses (U×Iwc; U×Iwwc), indicating that *w*Ori does neither contribute to induce a higher level of CI nor induce additive effects on CI expression.

### Effects of Double-infection and Multiple-infection on Host Reproduction

The two groups of crosses in which CI induced by the double-infection and multiple-infection were detected (U×Iwc and U×Iwwc) showed a strong reduction in egg hatchability and a strongly male-biased sex ratio. In the predicted incompatible cross (♀U×♂Iwc), on average, (26.02±3.94)% of all eggs hatched, against 93.5–93.8% in the other crosses (Fig.1.C). Similarly, the mean egg hatchability between uninfected females and *multiple*-infected males (♀U×♂Iwwc) was (23.84±4.63)%, significantly lower than that found in compatible crosses (93.2%–96.7%) (Fig.1.E). The sex ratios of the offspring that did hatch in these two incompatible crosses were significantly lower than the sex ratios from the compatible crosses (Fig.1.D, F). These crosses displayed a high level of CI expression. All mites used in this research were infected and uninfected naturally, without any antibiotic treatment for exclusion of extra interferential factors. We were unable to find any individuals singly infected with *Wolbachia* or *Cardinium*, which made it difficult to evaluate the effects. All that we can say from these limited results is that the strength of CI induced by *Wolbachia* and *Cardinium* together is respectively strong. From the results of bacteria measurements, we found that the density of *w*Con was close to the density of *Cardinium*, due to which we infer that both *w*Con and *Cardinium* contribute to the strong CI.

### Effects of *w*Ori, *w*Con and *Cardinium* Infection on Host Fecundity

Fecundity of uninfected, single-infected and super-infected females was compared by analyzing the number of eggs laid in the first 5 days (Table.1). From these crosses we can draw three conclusions: (i) Mating with uninfected males increases the fecundity of double-infected females, which consequently promotes the spread of *w*Con and *Cardinium*. (ii) The fecundity of multiple-infected females can be increased by mating with uninfected males, but decreased by mating with double-infected males. It means that double-infected males act as a block to expansion of multiple infection by weakening the fecundity of multiple-infected females. Based on this point, we agree with your opinion that there is a competition between the doubly and triply infected females. (iii) Both the double-infected and multiple-infected males strongly depress the fecundity of their uninfected mates. In particular, multiple-infected males caused an even greater depression of fecundity.

### Bacteria Density Measurement

**Figure 2:**
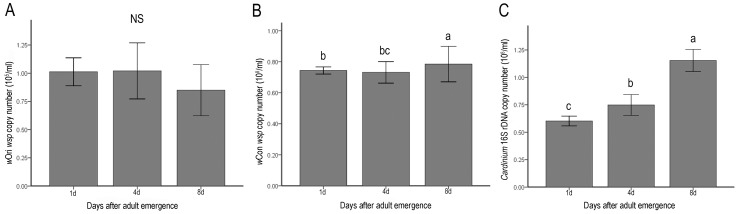
Mean densities of *w*Ori (A), *w*Con (B) and *Cardinium* (C) during aging of multiple-infected males in *T. phaselus* population. Copy numbers per ml were determined by quantitative PCR using the *w*Ori wsp gene, *w*Con *wsp* gene and 16S rDNA gene (error bars represent 95% bootstrap confidence intervals, n = 8 per each column). a, b and c represent statistic groups (Tukey-HSD test, p<0.05); NS, not significant at the 5% level.

To understand why multiple-infected males were unable to induce a higher level of CI, we examined the infection densities of *w*Ori, *w*Con and *Cardinium* in the double- and multiple-infected individuals, in which the CI strength seemed to be related to the bacteria density. Our hunch proved to be correct: *w*Ori was present at a rather low density in the multiple-infected males, being only 0.1%–0. 2% of the densities of *w*Con and *Cardinium* (Fig.2).

**Figure 3:**
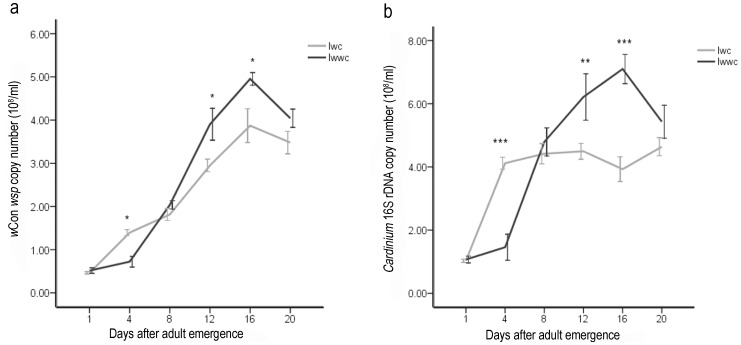
Comparisons of *w*Con (a) and *Cardinium* (b) densities between double-infected and multiple-infected females in *T. phaselus* population. Copy numbers per ml were determined by quantitative PCR using the *w*Con *wsp* gene and 16S rDNA gene (error bars represent 95% bootstrap confidence intervals, n = 8 per each column). *P<0.05; **P<0.01; ***P<0.001.

In order to reveal whether the existence of *w*Ori affects the proliferation of *w*Con and *Cardinium*, we compared the *w*Con and *Cardinium* densities in double- and multiple-infected females separately (Fig.3).The double-infected females have higher *w*Con and *Cardinium* copy numbers than the multiple-infected females when they were 4 days old. For the older females (from 8 days old to 20 days old), infection with *w*Ori enhanced the proliferation of *w*Con and *Cardinium*. Furthermore, the dynamic of *w*Con density during host aging are affected by *w*Ori infection. *w*Con density increased more rapidly in multiple-infected females (from (0.40±0.04)×10^8^/ml to (4.95±0.14) ×10^8^/ml) than in double-infected females (from (0.46±0.03)×10^8^/ml to (3.87±0.39) ×10^8^/ml) during the first 16 days of their life spans (Fig.3.a). In addition, there are different dynamics of *Cardinium* density in double-infected females and multiple-infected females. The density of *Cardinium* in double-infected females was (1.03±0.05)×10^8^/ml on the first day post eclosion (Fig.3.b). However, from the fourth day to the twentieth day, the density fluctuated between (3.93±0.39)×10^8^ and (4.64±0.29)×10^8^ copies per ml (Fig.3b). During this period, Mann-Whitney tests indicated that the differences between each pair of two ages were not significant (*P*>0.05). However, in multiple-infected females, *Cardinium* density rose from (1.07±0.11)×10^8^/ml to (7.10±0.46) ×10^8^/ml step by step from the first day to the sixteenth day post eclosion, and then, it fells to (5.43±0.52)×10^8^/ml on the twentieth day. Mann-Whitney tests indicated that the differences between each pair of two ages were significant (*P*<0.05).

### Survival Assessment

**Figure 4:**
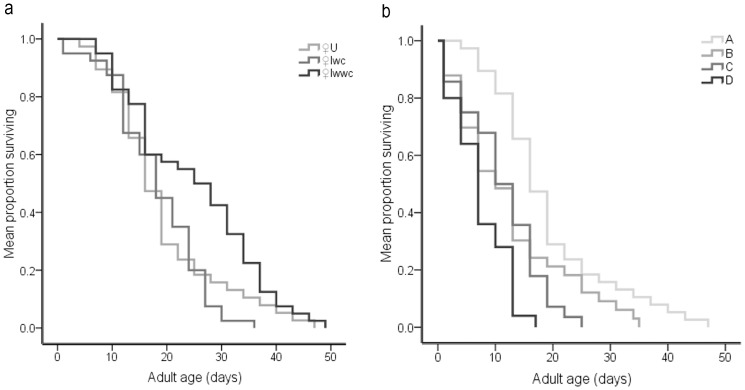
Survival curves of *T. phaselus* females. (a) Survival curves of females with different infection statuses; (b) Survival curves of uninfected females with different mating statuses: A : Virgin females; B : females mated with uninfected males; C : females mated with double-infected males; D:females mated with multiple-infected males.

To discover the effects that *w*Ori had on its female hosts, we measured the longevities of uninfected (U), double-infected (Iwc) and multiple-infected (Iwwc) females. Because of the variously uncertain factors in the environment, it is almost impossible to obtain the natural dynamics of the longevity of the hosts on different infection statuses. In order to exclude any influence of differences in environmental conditions, we measured the females on different infection statuses under ideal conditions, including no predation, no food limitation and no mating behavior. On ideal conditions (no predation, no food limitation and no mating behavior), we found that *w*Ori infection prolonged female survival (Fig.4.a). This means that the longevity of multiple-infected females (25.08±1.88 days) was longer than the longevities of double-infected females (18.35±1.26 days) and uninfected females (19.62±1.65 days; x^2^ = 13.43, *df* = 1, *P*<0.001; x^2^ = 3.894, *df* = 1, *P* = 0.048). Consequently, *w*Ori can remain longer in the female hosts, which allows it to be transmitted to more offspring. We regard this phenomenon as a force causing *w*Ori to persist in this population under ideal conditions.

To determine the effect of infection status of males on the longevity of females, we compared the longevities of different groups of uninfected females which are virgins, and those separately mated with uninfected, double-infected and multiple-infected males. From the survival curves of these four groups of females (Fig.4.b), three conclusions can be drawn. First, virgin females (line A, 19.62±1.65 days) have a longevity advantage over mated females (line B, 12.49±1.73 days; line C, 11.29±1.28 days; line D, 7.40±0.95 days). This means that mating behavior is a direct factor that shortens the females’ lifespan. Second, no significant difference was found between the survival curves of females (line B, 12.49±1.73 days) mated with uninfected males and females (line C, 11.29±1.28 days) mated with double-infected males (x^2^ = 0.696, *df* = 1, *P* = 0.404). Third, mating with multiple-infected males dramatically shortened the life span of uninfected females (line D, 7.40±0.95 days). The difference in longevity between females mated with uninfected males and females mated with multiple-infected males was significant (x^2^ = 5.015, *df* = 1, *P* = 0.025). Also, females mated with multiple-infected males have a significantly shorter life span than females mated with double-infected males (x^2^ = 6.398, *df* = 1, *P* = 0.011). These results clearly show that the longevity of uninfected females mated with multiple-infected males was further shortened by not only mating behavior, but also the spermatozoa modified with *w*Ori. The ability of *w*Ori to reduce the longevity of uninfected females by their spermatozoa may act as an additional force to drive the spread of *w*Ori through a mite population. This effect of *w*Ori gives extra strength to a multiple-infection to invade an uninfected population.

## Discussion

To our knowledge, this is the first case of multiple infection with *Cardinium* and two strains of *Wolbachia* in arthropods. A possible mechanism of the multiple infections in the wild is horizontal transmission. An infection survey of wild spider mite populations in Zhejiang Province shows that *Tetranychus truncatus*, another important agriculture pest co-existing in this area, is singly infected by *w*Ori (own unpublished data). Probably, *w*Ori in *T. phaselus* was acquired through horizontal transmission from *T. trunctatus*. It could also be horizontal transmitted from *T. phaselus* to *T. trunctatus*. Another possibility is that both species could have inherited the triple infection from a common ancestor and *Cardinium* and *w*Con became lost in *T. trunctatus* [31], [32], [33]. However, further study is needed to test this hypothesis.

The results of bacteria density measurements and crossing experiments appear to show there is a reciprocal relationship between *w*Ori and the other bacteria (*w*Con and *Cardinium*) in the same host. *w*Con and *Cardinium* share their intensive CI with *w*Ori, although *w*Con and *Cardinium* are dominant strains over *w*Ori, which compensates for the CI absence of *w*Ori. Even though *w*Ori suppressed the densities of *w*Con and *Cardinium* in the four-day- old multiple-infected females because of the limited nutrients in the young hosts, it had favorable effects on the densities of *w*Con and *Cardinium* in the older hosts (Fig. 3). During the medium-term of their life span (from the eighth day to the twentieth day), the main oviposition period of the spider mite, *w*Ori significantly facilitated the proliferation of *w*Con and *Cardinium*. That means more *w*Con and *Cardinium* can be transmitted to offspring in multiple-infected females than in double-infected females. In general, the reciprocal relationship allows the three strains of bacteria to co-exist in the same host.

The powerful spreading capability of *Wolbachia* has attracted considerable attention as a potential gene driving system. The expression of transgenes could be simplified by exploiting the population invasion ability of *Wolbachia*. Within this theoretical framework, the capability of *Wolbachia* to invade and maintain themselves in host populations depends on three main parameters: (*i*) the strength of CI, (*ii*) maternal transmission efficiency, and (*iii*) fitness effects on the host [34].

An empirical study directly examined the transmission efficiency of *Wolbachia* in spider mites in the laboratory [35], during which a perfect maternal transmission has been found. On this basis, we infer that transmission efficiency of *w*Ori is perfect probably, ensuring its maintenance in the *T. phaselus* population. However, future experiments are needed to examine our conjecture.

Cytoplasmic incompatibility (CI) is most commonly associated with *Wolbachia* infections. The intensity of CI is an important parameter that determines the spread and maintenance of *Wolbachia* infection in the host population [36]. CI results in *Wolbachia* spread, as witnessed in nature in *Drosophila simulans* [10] and in a laboratory population of *Wolbachia*-transinfected *A. aegypti* [37]. Some strains of *Wolbachia* induce high CI, which leads to rapid population invasion. For instance, the *Wolbachia* strain *w*Ri has been shown to spread geographically at a rate of 100 km/year into uninfected *D. simulans* populations through the action of CI [11].

*Wolbachia* super-infections (that is co-infection with two or more *Wolbachia* strains) occur naturally [14], [22], [38] and have been generated artificially as well [39], [40]. These super-infections typically have additive effects, such that a super-infected male is unidirectionally incompatible with both single-infected and uninfected females, whereas super-infected females can rescue infections in both super-infected and single-infected females. In the present study, superinfection with *w*Ori did not induce a higher level of CI, indicating that *w*Ori did not have an additive effect. The bacteria density measurement**s** lend qualified support to the hypothesis that *w*Con and *Cardinium* interfere with the ability of *w*Ori to cause CI by suppressing *w*Ori densities. *w*Ori was present in the lowest copy number among those bacteria in males, being only about 0.1%–0.2% of the densities of *w*Con and *Cardinium*. Obviously, *w*Con and *Cardinium* are dominant species compared with *w*Ori. Similarly, suppression of the density of one symbiont by the other has been found before in natural infections [41], [42], and density differences may also have been the reason for the lack of CI modification and rescue in the *Wolbachia* super-infection created in mosquitoes by Walker *et al* [40]. Moreover, studies of the interaction of *Wolbachia* with developing sperm show that an abundance of *Wolbachia* in the testes is required to induce CI. We hypothesized that the testes of *T. phaselus* have been occupied by *w*Con and *Cardinium*, leaving limited room for *w*Ori. This is a potential mechanism of undetectable CI of *w*Ori, which could be further proved by localizing the symbionts in testes of multiple-infected males [43], [44]. In this study, the interactions between *w*Con and *Cardinium* could not be investigated because of the absence of lines of singly infected with *w*Con or *Cardinium*.

Several strains of *Wolbachia*, such as *w*Au from *D. simulans* and *w*Mel from *D. melanogaster,* and *w*Stri in the brown planthopper *Nilaparvata lugens* [15], [45], have invaded natural populations even though they induce little to no CI under field conditions. It has been hypothesized that these strains provide an as yet undermined fitness benefit to their hosts. Our results provide important evidence for this hypothesis. The longer-lived multiple-infected females are conducive to the spread of *w*Ori, because they have a greater number of offspring. In addition, the non-CI *w*Ori appears to have a novel mechanism for invading and diffusing through a population. The survival curves of uninfected females indicate that the shorter life span of uninfected females induced by mating with multiple-infected males acts to spread *w*Ori specifically in multiple-infected individuals. This is because the multiple-infected spermatozoa, which decrease the longevity of uninfected females, help to decrease the number of uninfected offspring. We conjecture that the ability of reduction of the longevity of uninfected females probably acts as a new and specific force for *w*Ori to invade and spread through this spider mite population. In addition, fecundity of multiple-infected females affects the survival success of *w*Ori in a similar way. Both the multiple-infected and double-infected males can suppress the fecundity of uninfected females. Although the multiple-infected males are stronger than double-infected males in depressing the fecundity of uninfected females, it is uncertain whether multiple-infection can succeed in the competition with double-infection.

Several studies have proved that the spread of bacterial symbionts can be driven by various host fitness benefits. Dobson *et al* [14] showed that the combination of CI and host fecundity increase can reduce the threshold infection frequency required for *Wolbachia* invasion and accelerate the rate of *Wolbachia* invasion in the super-infected *A. albopictus* population. In contrast, a higher threshold infection frequency and slower replacement rate is shown in the single-infected *A. albopictus* population, since it is driven only by CI. In addition, the rapid spread of *Rickettsia* sp. nr. *bellii* in a population of an invasive agricultural pest, the sweet potato whitefly, *Bemisia tabaci* is driven by fitness benefits and female bias [46]. These results support our hypothesis that the spread of *w*Ori can be driven by the combination of CI shared with *w*Con and *Cardinium*, host longevity increase and attenuating effects on fecundity and longevity of uninfected females.

In conclusion, we describe novel forces driving the spread of *Wolbachia*. The new forces we found may provide insights into the distribution of *Wolbachia* bacteria among species. In addition, the new associations of inherited *w*Ori and hosts that have been regarded as forces driving the spread of the symbiont may result in rapid evolution of both partners and phenotypic shifts that optimize the symbiosis [47]. They can also be exploited to introduce desirable traits in to arthropod hosts using *Wolbachia* [12], [48].
